# Physical Properties and Photovoltaic Application of Semiconducting Pd_2_Se_3_ Monolayer

**DOI:** 10.3390/nano8100832

**Published:** 2018-10-14

**Authors:** Xiaoyin Li, Shunhong Zhang, Yaguang Guo, Fancy Qian Wang, Qian Wang

**Affiliations:** 1 Center for Applied Physics and Technology, HEDPS, Department of Materials Science and Engineering, College of Engineering, Peking University, Beijing 100871, China; lixiaoyin@pku.edu.cn (X.L.); guoyaguang@pku.edu.cn (Y.G.); qianwang7@pku.edu.cn (F.Q.W.); 2 Collaborative Innovation Center of IFSA (CICIFSA), Shanghai Jiao Tong University, Shanghai 200240, China; 3 Institute for Advanced Study, Tsinghua University, Beijing 100084, China; zhangshunhong@tsinghua.edu.cn

**Keywords:** palladium selenide monolayer, physical properties, light-harvesting performance, type-II heterostructure, first principles calculations

## Abstract

Palladium selenides have attracted considerable attention because of their intriguing properties and wide applications. Motivated by the successful synthesis of Pd_2_Se_3_ monolayer (Lin et al., Phys. Rev. Lett., 2017, 119, 016101), here we systematically study its physical properties and device applications using state-of-the-art first principles calculations. We demonstrate that the Pd_2_Se_3_ monolayer has a desirable quasi-direct band gap (1.39 eV) for light absorption, a high electron mobility (140.4 cm^2^V^−1^s^−1^) and strong optical absorption (~10^5^ cm^−1^) in the visible solar spectrum, showing a great potential for absorber material in ultrathin photovoltaic devices. Furthermore, its bandgap can be tuned by applying biaxial strain, changing from indirect to direct. Equally important, replacing Se with S results in a stable Pd_2_S_3_ monolayer that can form a type-II heterostructure with the Pd_2_Se_3_ monolayer by vertically stacking them together. The power conversion efficiency (PCE) of the heterostructure-based solar cell reaches 20%, higher than that of MoS_2_/MoSe_2_ solar cell. Our study would motivate experimental efforts in achieving Pd_2_Se_3_ monolayer-based heterostructures for new efficient photovoltaic devices.

## 1. Introduction

Two-dimensional (2D) transition metal chalcogenides (TMCs), including semiconducting MoS_2_ [1], MoSe_2_ [2], WS_2_ [3], WSe_2_ [4], ReS_2_ [5], PtS_2_ [6], PdSe_2_ [7,8], and metallic VS_2_ [9] and NbS_2_ [10] are of current interest because of their extraordinary properties and practical applications in catalysis [11], electronics [12,13,14], optoelectronics [15,16] and valleytronics [17,18]. Among them, the layered PdSe_2_ has attracted special attention due to its unique atomic configuration and electronic properties [8,19,20]. Whereas previous studies mainly focused on the PdSe_2_ monolayer that has the same structural form as a single layer of the bulk PdSe_2_ [7,21,22]. Very recently, Lin et al. reported the successful exfoliation of a new monolayer phase with a stoichiometry of Pd_2_Se_3_ [23], and found that Se vacancies in the pristine PdSe_2_ reduce the distance between the layers, melding the two layers into one, thus, resulting in the formation of the Pd_2_Se_3_ monolayer. Due to its structural novelty, subsequent efforts have been made to further explore this new material, including its electronic and optical properties [24] and thermoelectric performance [25], as well as theoretical calculations and experimental synthesis of the lateral junctions between a PdSe_2_ bilayer and the Pd_2_Se_3_ monolayer [26].

We noticed that in Reference [24] the results were obtained from standard density functional theory (DFT) calculations (the Perdew-Burke-Ernzerhof (PBE) functional [27] for the generalized gradient approximation (GGA)), which is well-known to underestimate the electronic band gap of semiconductors. However, the accurate description of electronic structure is important for further investigation of electronic and optical properties. To overcome this limitation, various theoretical approaches have been developed. Among them, the hybrid functional that combines standard DFT with Hartree-Fock (HF) calculations has been widely used for calculating the band gaps, because it predicts more reliable physical properties and keeps a good compromise with computational efficiency. Therefore, we use the Heyd-Scuseria-Ernzerhof (HSE06) hybrid functional [28,29] to study the electronic, transport and optical properties of the newly synthesized Pd_2_Se_3_ monolayer. We show that this monolayer possesses a desirable bandgap for light harvesting, offering better opportunity for photovoltaic applications. Moreover, its electronic structure can be effectively tuned by applying biaxial strain, and indirect to direct bandgap transition occurs with a small critical strain of 2%. In addition, a stable Pd_2_S_3_ monolayer can be formed by substituting Se with S, which can be used to construct a type-II heterostructure with the Pd_2_Se_3_ monolayer. The heterostructure-based solar cell can reach a high power conversion efficiency (PCE) of 20%. These fascinating properties make the Pd_2_Se_3_ monolayer a promising candidate for future applications in nanoscale electronics and photonics.

## 2. Computational Methods 

Within the framework of DFT, our first-principles calculations are performed using the projector augmented wave (PAW) method [30] as implemented in the Vienna Ab initio Simulation Package (VASP) [31]. The Perdew-Burke-Ernzerhof (PBE) functional [27] with the generalized gradient approximation (GGA) is used to treat the electron exchange-correlation interactions in crystal structure calculations, while the Heyd-Scuseria-Ernzerhof (HSE06) hybrid functional [28,29], which includes the Hartree-Fock exchange energy and the Coulomb screening effect, is used to calculate the electronic and optical properties. A kinetic energy cutoff of 350 eV is set for the plane wave basis. The convergence criteria are 10^−5^ eV and 10^−3^ eV/Å for total energy and atomic force components, respectively. The Brillouin zone is represented by *k* points with a grid density of 2π × 0.02 Å^−1^ in the reciprocal space using the Monkhorst-Pack scheme [32]. An adequate vacuum space (~20 Å) in the direction perpendicular to the sheet is used to minimize the interlayer interactions under the periodic boundary condition. Spin-orbit coupling (SOC) interactions are not included since our calculation shows that the SOC has negligible effect on electronic structure of the monolayer (see Figure S2). Phonon dispersion and density of states (DOS) are calculated using the finite displacement method [33] as implemented in the Phonopy code [34].

In the calculation of carrier mobility (*µ*), we consider the perfect crystal of the monolayer without defects and impurities. In addition, carrier mobility is a function of temperature. We set the temperature to be 300 K in our calculation, since most devices work at room temperature. In this situation, the dominant source of electron scattering is from acoustic phonons and the carrier mobility can be obtained using deformation potential theory proposed by Bardeen and Shockley [35], which has been successfully employed in many 2D materials [36,37,38,39]. Using effective mass approximation, the analytical expression of carrier mobility in 2D materials can be written as

(1) $$\mu =\frac{e\hbar^{3}C}{k_{B}Tm^{*}m_{d}E_{1}^{2}}\;$$

*C* is the elastic modulus of the 2D sheet, *T* is the temperature, which is taken to be 300 K in our calculations, m* = *ħ*^2^[∂^2^*E*(*k*)/∂*k*^2^]^−1^ is the effective mass of the band edge carrier along the transport direction and *m_d_* is the average effective mass determined by $m_{d}=\sqrt{m_{x}^{*}m_{y}^{*}}$. *E*_1_ is the DP constant defined as the energy shift of the band edge with respect to lattice dilation and compression, and *k*_B_ and *ħ* are Boltzmann and reduced Planck constants, respectively.

The optical absorption coefficient (*α*) can be expressed as [40,41,42]

(2) $$\alpha (\omega )=\sqrt{2}\omega {\left[\sqrt{\varepsilon_{1}^{2}(\omega )+\varepsilon_{2}^{2}(\omega )}-\varepsilon_{1}(\omega )\right]}^{1/2}\;$$

where *ε*_1_(*ω*) and *ε*_2_(*ω*) are the real and imaginary parts of the frequency-dependent dielectric functions which are obtained using the time-dependent Hartree-Fock approach (TDHF) based on the HSE06 hybrid functional calculations [43]. The model, developed by Scharber et al*.* for organic solar cells [44] and exciton-based 2D solar cells [45,46,47,48,49], is used to calculate the maximum PCE in the limit of 100% external quantum efficiency (EQE), which can be written as

(3) $$\eta =\frac{\beta_{FF}V_{oc}J_{sc}}{P_{solar}}=\frac{0.65(E_{g}^{d}-\Delta E_{c}-0.3)\int_{E_{g}^{d}}^{\infty }\frac{P(\hbar \varpi )}{\hbar \varpi }d(\hbar \varpi )}{\int_{0}^{\infty }P(\hbar \varpi )d(\hbar \varpi )}\;$$

Here, the fill factor $\beta_{FF}$, which is the ratio of maximum power output to the product of the open-circuit voltage (*V_oc_*) and the short-circuit current (*J_sc_*), is estimated to be 0.65 in this model. *V_oc_* (in eV) is estimated by the term ($E_{g}^{d}-\Delta E_{c}-0.3$), where $E_{g}^{d}$ is the bandgap of the donor and $\Delta E_{c}$ is the conduction band (CB) offset between donor and acceptor. *J_sc_* is obtained by $\int_{E_{g}^{d}}^{\infty }\frac{P\left(\hbar \varpi \right)}{\hbar \varpi }d\left(\hbar \varpi \right)$, and the total incident solar power per unit area *P_solar_* is equal to $\int_{0}^{\infty }P(\hbar \varpi )d(\hbar \varpi )$. Here, *ħ* and *ϖ* are reduced Planck constants and photon frequency, and *P*(*ℏϖ*) is the air mass (AM) 1.5 solar energy flux (expressed in W m^−2^ eV^−1^) at the photon energy (*ℏϖ*).

## 3. Results and Discussion

### 3.1. Geometric Structure of Pd_2_Se_3_

**Figure 1:**
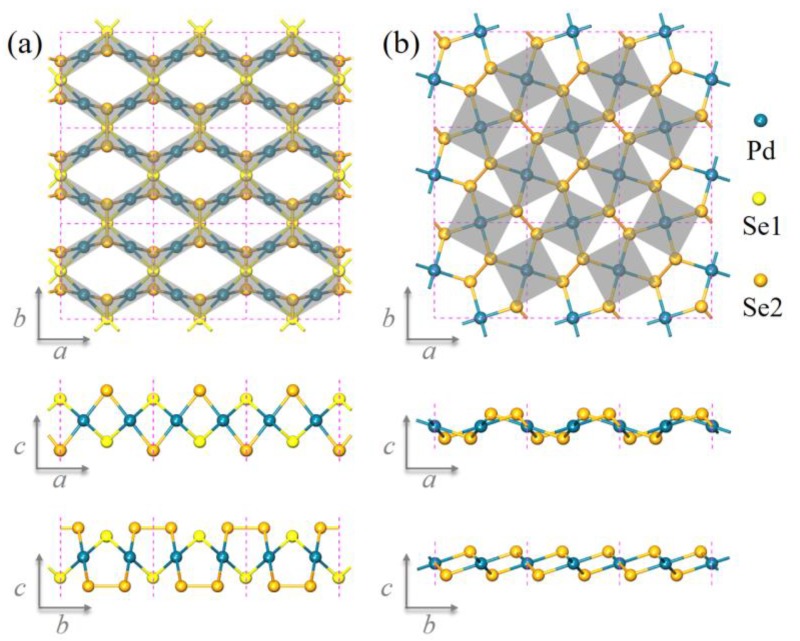
Optimized atomic structure of (**a**) Pd_2_Se_3_, and (**b**) PdSe_2_ monolayers. The gray tetragons and purple dashed rectangles correspond to the planar (PdSe_4_) units and the primitive cells of the two structures, respectively.

Figure 1a,b shows the optimized monolayer structures of Pd_2_Se_3_ and PdSe_2_ respectively (the structural details are listed in Table S1, Supplementary Materials). For simplicity, we refer these two monolayer structures as Pd_2_Se_3_ and PdSe_2_ in the following discussions unless stated otherwise. There are similarities as well as differences between the two structures. On one hand, they both consist of a layer of metal Pd atoms sandwiched between the two layers of chalcogen Se atoms, and each Pd atom binds to four Se atoms forming the square-planar (PdSe_4_) structural units. On the other hand, Pd_2_Se_3_ indeed distinguishes itself from PdSe_2_ in the following characteristics: (1) Pd_2_Se_3_ possesses *Pmmn* symmetry (point group *D_2h_*) with four Pd and six Se atoms in one unit cell. While the symmetry of PdSe_2_ is *P*2_1_/*c* (point group *C_2h_*) and each unit cell contains two Pd and four Se atoms. The different crystal symmetries result in distinct resonance in the Raman spectroscopy, which can serve as an efficient and straightforward clue for experimentalists to confirm the formation of Pd_2_Se_3_. The details about the calculated Raman spectra of Pd_2_Se_3_ and PdSe_2_ are presented in the Supporting Information (Figure S1). (2) There are two chemically nonequivalent Se in Pd_2_Se_3_, marked in orange (Se2) and yellow (Se1) respectively. The two neighboring Se2 atoms form a covalent Se-Se bond while each Se1 atom is unpaired and binds to four neighboring Pd atoms. Whereas in PdSe_2_, all Se atoms are in dimers and form the Se-Se bonds. (3) In Pd_2_Se_3_, the Se-Se dumbbells are parallel to the Pd layer, while in PdSe_2_, they cross the Pd layer. (4) Pd_2_Se_3_ and PdSe_2_ have different charge-balanced formulas, written as (Pd^2+^)_2_(Se^2−^)(Se_2_^2−^) and (Pd^2+^)(Se_2_^2−^) respectively, due to the different chemical environments of Se atoms in the two structures. Since the properties of materials are essentially determined by their geometric structures, one can expect that Pd_2_Se_3_ would possess some new and different properties from those of PdSe_2_.

### 3.2. Electronic Properties of Pd_2_Se_3_

**Figure 2:**
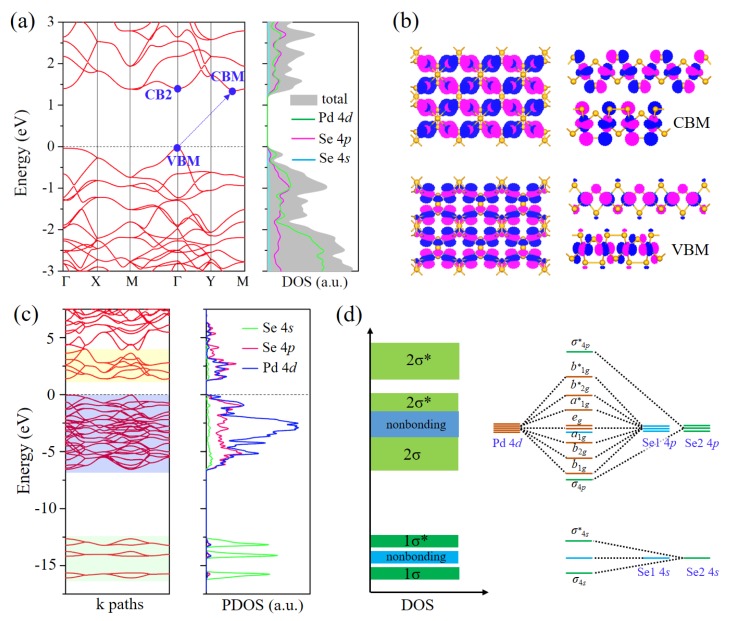
(**a**) Band structure and DOS around the Fermi level. The VBM and CB(M) are marked by blue dots; (**b**) Spatial visualization of wave functions for the VBM and CBM, using an isosurface of 0.04 eÅ^−3^; (**c**) Band structure and partial DOS with all valence states included. (**d**) Schematics of DOS and energy level diagram.

We then investigated the electronic properties of Pd_2_Se_3_ by calculating its electronic band structure and density of states (DOS) using the hybrid HSE06 functional. Figure 2a shows the calculated band structure around the Fermi level and corresponding total and partial DOS. The bandgap size of Pd_2_Se_3_ is 1.39 eV, close to the optimum value (~1.3 eV) for solar cell materials [50,51,52]. Although Pd_2_Se_3_ is an indirect bandgap semiconductor with the valence band maximum (VBM) located at the Γ point and the conduction band minimum (CBM) located on the Y-M path, Pd_2_Se_3_ can be considered as a quasi-direct bandgap semiconductor because of the existence of the sub-CBM at the Γ point (CB2) that is only marginally higher in energy than the true CBM (the energy difference is less than 50 meV). The weakly indirect bandgap is desirable for photovoltaic applications since it can simultaneously increase optical absorbance and photocarrier lifetimes [41,53,54]. To assess the effect of SOC interaction, we computed the band structure of Pd_2_Se_3_ at the level of HSE06+SOC. The results in Figure S2 reveal that the SOC in Pd_2_Se_3_ is weak and has negligible effect on the bandgap of this structure. Hereafter, we do not include the SOC interaction and just use the HSE06 scheme for calculations in this study.

An analysis of the partial DOS in Figure 2a indicates that the electronic states of valance and conduction bands mainly originate from Se 4*p* and Pd 4*d* orbitals. In addition, the overlap of the orbital-projected DOS implies strong hybridization, that is, the formation of covalent bonds between Se 4*p* and Pd 4*d* orbitals. By calculating wave functions for the VBM and CBM, we visualized their electronic states showing distinct antibonding features for both of them (see Figure 2b). However, to gain a better understanding for the covalent bonding in this 2D structure, the electronic bands not only limited to near the Fermi level but also in a large energy range should be taken into account.

Figure 2c displays the band structure and partial DOS including all occupied and sufficient unoccupied states of Pd_2_Se_3_. Combining crystal field theory and crystal structure chemistry analysis, we can clearly identify the electronic states in the energy range from −17 to 4 eV. From partial DOS, the bands in the energy range of −17 ~ −12.5 eV are primarily from Se 4*s* orbitals. According to the different bonding states, they can be classified into three groups. The bottom and upper subsets correspond to the bonding and antibonding states dominated by the formation of Se-Se bonds, and the middle part is the nonbonding state of the unpaired Se 4*s* orbitals. When the energy goes up, there occurs Pd 4*d* orbitals. In Pd_2_Se_3_, the Pd atom is coordinated in a nearly perfect square-planar geometry, and its 4*d* orbitals split into four energy levels, i.e., *e_g_* (*d_xz_*/*d_yz_*), *a*_1*g*_ (*d_z2_*), *b*_2*g*_ (*d_xy_*), and *b*_1*g*_ (*d_x2-y2_*) from low to high energy. These *d* orbitals overlapping with Se 4*p* orbitals constitutes the bands in the energy range of −7.5 ~ 4 eV. In Figure 2d, we present the schematic drawing of DOS and energy level diagram of Pd_2_Se_3_ to explain details about how Pd 4*d* orbitals interact with Se 4*p* orbitals. It shows that *a*_1*g*_, *b*_2*g*_, and *b*_1*g*_ orbitals hybridize with Se 4*p* orbitals leading to lower energy bonding states and higher energy antibonding states, whereas the nonbonding states in the energy range from −4 to −1.7 eV stemming mainly from *e_g_* orbitals. More importantly, the bandgap that separates occupied and unoccupied states lies in the antibonding region and amounts to the splitting energy between and states, consistent with the results of wave functions for the VBM and CBM in Figure 2b. The systematic and deep exploration of electronic structure of Pd_2_Se­_3_ is crucial for understanding its properties and origins of intriguing physical phenomena.

### 3.3. Strain Engineering of Electronic Band Structure of Pd_2_Se_3_

From above electronic structure analysis, it is clear that Pd_2_Se_3_ is a covalent semiconductor, and a connection between elastic strain and its electronic structure is expected. This is because elastic strain generally weakens the covalent interaction as the bonds lengthen, exerting efficient modulation on the band energies and bandgap. For this reason, we applied a biaxial tensile strain to Pd_2_Se_3_ and study its effect on the electronic bands of Pd_2_Se_3_.

**Figure 3:**
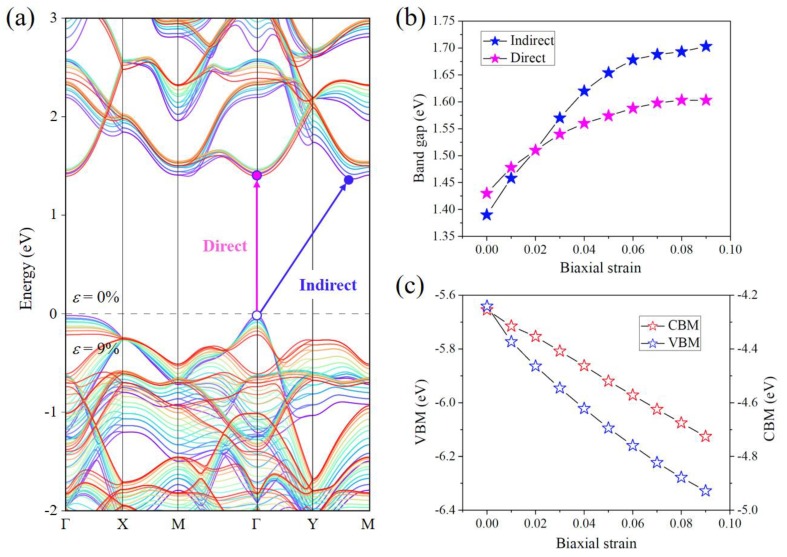
(**a**) Electronic band structure of the Pd_2_Se_3_ monolayer under biaxial strains varying from 0% (violet line) to 9% (red line); (**b**) Direct and indirect bandgaps under different biaxial strains; (**c**) Biaxial strain-dependent energies of the VBM and CBM with respect to the vacuum level. All calculations are based on the HSE06 functional.

Figure 3a shows the evolution of band structure with biaxial strain varying from 0% to 9%. It indicates that both direct and indirect bandgaps increase, and a transition from indirect bandgap to direct bandgap occurs when the biaxial strain is applied. To acquire a more accurate energy profile, we present the strain-dependent bandgaps in Figure 3b, which clearly shows the increasing trend of bandgaps and the bandgap transition from indirect to direct at the critical strain of 2%. 

The strain-dependent bandgap of Pd_2_Se_3_ can be understood by analyzing its electronic structure in Figure 2, which shows that the valence and conduction bands both originate from antibonding states. Application of a tensile strain increases the Pd–Se bond length thus decreases the amount of orbital overlap, leading to the stabilization of valence and conduction bands and reducing them in energy. This is consistent with our results, as shown in Figure 3c, which displays the strain-dependent energy levels for the VBM and CBM. However, since the biaxial tensile strain not only enlarges the bond length but also distorts the square-planar ligand field (see Figure S3 for details), the valence band responds more strongly to strains than the conduction band, resulting in the increase of bandgap in the imposed strain filed.

Additionally, we also examined the structural stability under biaxial strains. The phonon dispersion in Figure S4 demonstrates that the structure remains stable under the strain of 9%. The large strain tolerance and an electronic structure that has a continuous response in the imposed strain field indicate the great potential of Pd_2_Se_3_ in future flexible electronics.

### 3.4. Transport Properties of Pd_2_Se_3_

We also investigated the transport properties of Pd_2_Se_3_ by calculating its room-temperature carrier mobilities as summarized in Table 1. One can see that the mobilities for both electrons and holes are slightly anisotropic along the *x* and *y* directions, due to the structural anisotropy of Pd_2_Se_3_. Meanwhile, the electron mobility along the *y* direction is estimated to be 140.4 cm^2^V^−1^s^−1^, significantly higher than that of hole. When compared with PdSe_2_, whose carrier mobilities are calculated at the same theoretical level and listed in Table 1, Pd_2_Se_3_ possesses higher electron mobility and lower hole mobility, showing strong asymmetry in electron and hole transport. Although the carrier mobilities of Pd_2_Se_3_ is lower than the theoretical predicted carrier mobilities of some other 2D materials [36,38,55], it is still commendable if realized in practice [20].

### 3.5. Optical Properties of Pd_2_Se_3_

**Figure 4:**
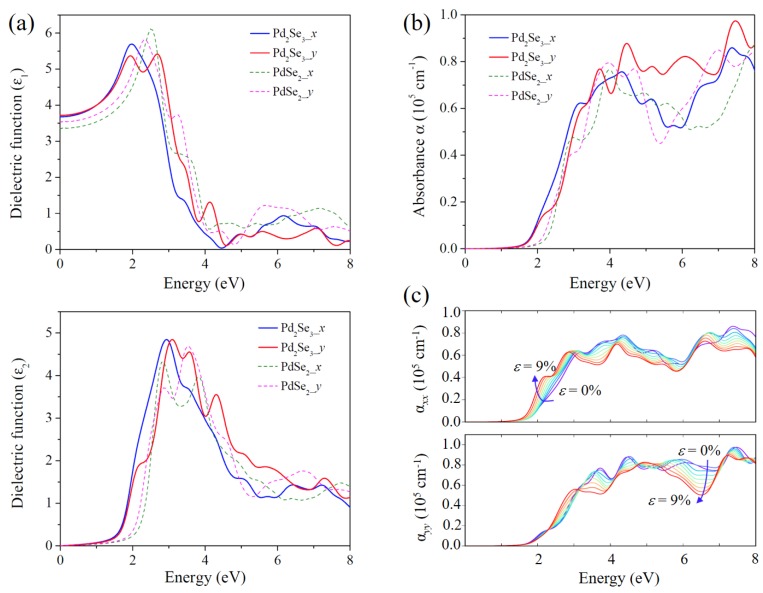
(**a**) Real part (*ε*_1_) and imaginary part (*ε*_2_) of the complex dielectric function, and (**b**) optical absorption spectra of Pd_2_Se_3_, as compared to those of PdSe_2_ along the *x* and *y* directions respectively; (**c**) Optical absorption spectra of Pd_2_Se_3_ under different biaxial strains from 0% (violet line) to 9% (red line).

Attracted by the suitable bandgap and intriguing electronic properties of Pd_2_Se_3_, we further explored its light-harvesting performance by calculating the dielectric functions based on the hybrid HSE06 functional. Figure 4a shows the real (*ε*_1_) and imaginary (*ε*_2_) parts of the frequency-dependent complex dielectric functions of Pd_2_Se_3_. With the dielectric functions, we derive its optical absorption coefficient (*α*), as shown in Figure 4b. For comparison, the absorption spectra of PdSe_2_ was also calculated. We notice that, for both Pd_2_Se_3_ and PdSe_2_, the overall absorption coefficients are close to the order of 10^5^ cm^−1^ and only show little difference along the *x* and *y* directions, which are considerably desirable for optical absorption. Moreover, as shown in Figure 4b, the absorption coefficient of Pd_2_Se_3_ is slightly larger than that of PdSe_2_ in nearly the entire of the energy range, indicating the improved light-harvesting performance of Pd_2_Se_3_ as compared with PdSe_2_. Furthermore, we also investigated the biaxial strain influence on the optical performance of Pd_2_Se_3_. The calculated strain-dependent optical absorption spectra are presented in Figure 4c. It shows that the strain slightly affects the optical absorption of Pd_2_Se_3_, which is favorable for applications in flexible systems since it guarantees steady performance of devices under stretching.

### 3.6. Extension and Photovoltaic Application of Pd_2_Se_3_

**Figure 5:**
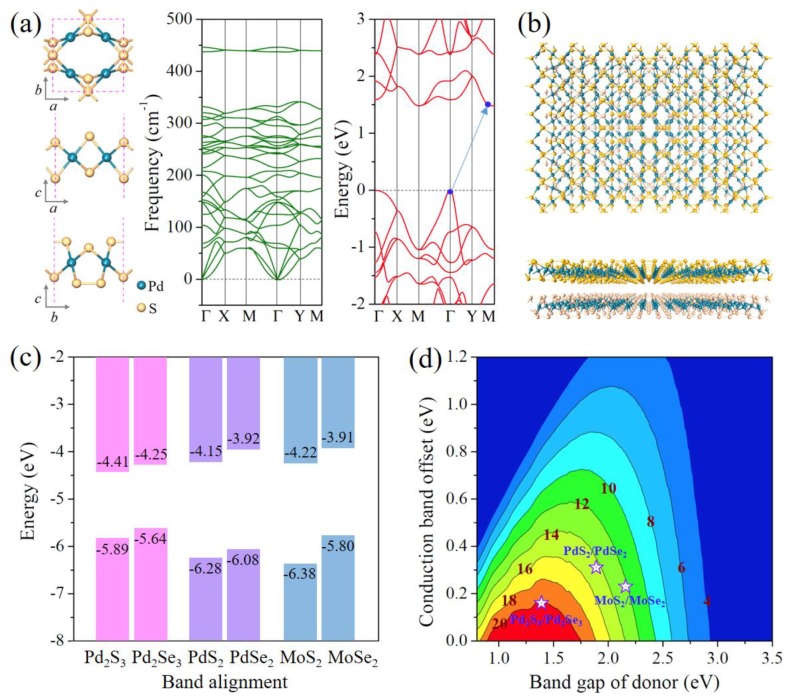
(**a**) Optimized atomic structure, phonon spectra and electronic band structure (at the HSE06 level) of the Pd_2_S_3_ monolayer; (**b**) Top and side views of the heterostructure composed of the Pd_2_S_3_ and Pd_2_Se_3_ monolayers; (**c**) Band alignments of the Pd_2_S_3_, Pd_2_Se_3_, PdS_2_, PdSe_2_, MoS_2_, and MoSe_2_ monolayers calculated using the HSE06 functional. The numbers are the CBM and VBM energies with respect to the vacuum level, which is set to zero when calculating the band alignment diagrams; (**d**) Computed PCE contour as a function of the donor bandgap and conduction band offset. Violet open stars mark the PCEs of Pd_2_S_3_/Pd_2_Se_3_, PdS_2_/PdSe_2_, and MoS_2_/MoSe_2_ heterostructure solar cells.

Moreover, we further explored the feasibility of other Pd_2_X_3_ monolayer phases, with X to be S and Te, respectively. Bulk PdS_2_ has the same geometrical structure as that of bulk PdSe_2_, thus the Pd_2_S_3_ monolayer might be experimentally synthesized following the same synthetic method as that of the Pd_2_Se_3_ monolayer. However, bulk PdTe_2_ prefers a 1T configuration, indicating that the Pd_2_Te_3_ monolayer might be inaccessible. To confirm our assumption, we calculated the phonon dispersions of the two structures. No imaginary mode exists in the phonon spectra of Pd_2_S_3_ (see Figure 5a), indicating its dynamical stability of the monolayer. Whereas the phonon spectra of the Pd_2_Te_3_ monolayer shows imaginary frequency near the Γ point (see Figure S5), demonstrating its structural instability. We then calculated the electronic band structure of the stable Pd_2_S_3_ monolayer (Figure 5a), verifying the feature of a semiconductor with an indirect bandgap of 1.48 eV.

Since 2D TMCs can be vertically stacked layer-by-layer forming the van der Waals heterostructures which can efficiently modulate properties of materials for applications in nanoscale electronic and photovoltaic devices, here we propose a van der Waals heterostructure composed of the Pd_2_Se_3_ and Pd_2_S_3_ monolayers (see Figure 5b) and study its interesting properties. A key indicator for heterostructures is the band alignment that defines the type of heterostructures. Thus, we calculated the band alignment of the Pd_2_Se_3_ and Pd_2_S_3_ monolayers, as shown in Figure 5c. One can see that the Pd_2_S_3_/Pd_2_Se_3_ heterostructure has a type-II (ladder) band alignment, which allows more efficient electron-hole separation for lighting harvesting. Such type-II heterostructure can be used as active materials in excitonic solar cells (XSCs) [46,47,48,49]. For the Pd_2_S_3_/Pd_2_Se_3_ heterostructure, the Pd_2_Se_3_ monolayer is the donor and the Pd­_2_S_3_ monolayer serves as the acceptor. With the approximation that the HSE06 bandgap equals optical bandgap and using the model developed by Scharber et al. [44], we obtained the upper limit of the PCE, reaching as high as 20% (Figure 5d). For comparison, we also calculated the band alignments for the PdS_2_/PdSe_2_ and MoS_2_/MoSe_2_ heterostructures, and find that they both are type-II heterostructures with predicted PCEs to be 14% and 12% respectively. The high PCE of the Pd_2_S_3_/Pd_2_Se_3_ heterostructure renders it a promising candidate in flexible optoelectronic and photovoltaic devices.

## 4. Conclusions

In summary, on the basis of DFT calculations, we systematically studied the properties and potential applications of the recently synthesized Pd_2_Se_3_ monolayer by focusing on its geometric structure, electronic band structure, and optical adsorption. Comparing with the previously reported PdSe_2_ monolayer, we found that the Pd_2_Se_3_ monolayer has the following merits: (1) A suitable quasi-direct bandgap (1.39 eV) for light absorption, (2) a higher electron mobility (140.4 cm^2^V^−1^s^−1^) and (3) a stronger optical absorption (~ 10^5^ cm^−1^) in the visible solar spectrum, showing promise of Pd_2_Se_3_ as an absorber material for future ultrathin photovoltaic devices. In addition, the Pd_2_Se_3_ monolayer combining with the stable Pd_2_S_3_ monolayer can form a type-II heterostructure, and the heterostructure solar cell system can achieve a 20% PCE. These findings would encourage experimentalists to devote more effort in developing Pd_2_Se_3_-based devices with high performance. 

## Figures and Tables

**Table 1:** Calculated deformation potential constant (*E*_1_), elastic modulus (*C*), effective mass (m^*^), and mobility (*μ*) for electron and hole in the *x* and *y* directions for Pd_2_Se_3_ and PdSe_2_ monolayers at 300 K.

	Carrier Type	*E*_1_ (eV)	*C* (N/m)	m^*^ (*m*_e_)	μ (cm^2^V^−1^s^−1^)
Pd_2_Se_3_	electron (*x*)	3.756	33.02	0.762	101.9
	electron (*y*)	3.785	32.93	0.543	140.4
	hole (*x*)	2.870	33.02	9.029	7.3
	hole (*y*)	12.082	32.93	0.187	19.9
PdSe_2_	electron (*x*)	9.542	32.45	0.429	43.36
	electron (*y*)	9.982	55.05	0.390	73.99
	hole (*x*)	3.352	32.45	0.656	97.94
	hole (*y*)	3.074	55.05	1.401	92.54

## Supplementary Materials

The following are available online at http://www.mdpi.com/2079-4991/8/10/832/s1, Table S1: Structural parameters of the Pd_2_Se_3_, Pd_2_S_3_, Pd_2_Te_3_, PdS_2_, PdSe_2_, MoS_2_, and MoSe_2_ monolayers; Figure S1: (a) Raman spectra of PdSe_2_ and Pd_2_Se_3_ monolayers. (b) and (c) are the corresponding Raman-active vibrational modes of the two structures; Figure S2: Electronic band structure of the Pd_2_Se_3_ monolayer calculated at the HSE06 level with and without considering the SOC interaction; Figure S3: Geometric structure of the Pd_2_Se_3_ monolayer under 0% and 9% biaxial tensile strain; Figure S4: Phonon dispersion of the Pd_2_Se_3_ monolayer under 9% biaxial tensile strain; Figure S5: Phonon dispersion of the Pd_2_Te_3_ monolayer.
