# Supplementary material for: Physical Properties and Photovoltaic Application of Semiconducting Pd2Se3 Monolayer
Source: Nanomaterials (Basel). 2018 Oct 14;8(10):832. doi: 10.3390/nano8100832 (PMC6215269; doi:10.3390/nano8100832)
Supplement: Supplementary file 1 [file nanomaterials-08-00832-s001.pdf]

Supplementary Materials for

Physical Properties and Photovoltaic Application of Semiconducting Pd_2Se_3 Monolayer

Xiaoyin Li, Shunhong Zhang, Yaguang Guo, Fancy Qian Wang and Qian Wang*

Table S1. Structural parameters of the Pd_2Se_3, Pd_2S_3, Pd_2Te_3, PdS_2, $PdSe_2$, MoS_2, and $MoSe_2$ monolayers.

Structure	Space group	Lattice parameters	Wyckoff atomic positions
Pd_2Se_3	*Pmmn* (59)	*a* = 5.97 Å<br>*b* = 6.14 Å<br>*c* = 20 Å	Pd 4*d* (0.75, 0.25, 0.5)<br>Se 2*a* (0, 0, 0.43163)<br>Se 4*e* (0, 0.19589, 0.59602)
Pd_2S_3	*Pmmn* (59)	*a* = 5.77 Å<br>*b* = 5.92 Å<br>*c* = 20 Å	Pd 4*d* (0.75, 0.25, 0.5)<br>S 2*b* (0, 0.5, 0.56447)<br>S 4*e* (0, 0.67633, 0.40947)
Pd_2Te_3	*Pmmn* (59)	*a* = 6.11 Å<br>*b* = 6.62 Å<br>*c* = 20 Å	Pd 4*d* (0.75, 0.25, 0.5)<br>Te 2*b* (0, 0.5, 0.57404)<br>Te 4*e* (0, 0.28901, 0.39424)
PdS_2	$P2_1/c$ (14)	*a* = 20 Å<br>*b* = 5.46 Å<br>*c* = 5.57 Å<br>β = 90º	Pd 2*b* (0.5, 0, 0)<br>S 4*e* (0.53177, 0.60415, 0.11104)
$PdSe_2$	$P2_1/c$ (14)	*a* = 20 Å<br>*b* = 5.74 Å<br>*c* = 5.91 Å<br>β = 90º	Pd 2*d* (0.5, 0.5, 0)<br>Se 4*e* (0.46268, 0.61049, 0.38002)
MoS_2	$P\bar{6}m2$ (187)	*a* = 3.18 Å<br>*b* = 3.18 Å<br>*c* = 20 Å	Mo 1*f* (0.33333, 0.66667, 0.5)<br>S 2*h* (0.66667, 0.33333, 0.42176)
$MoSe_2$	$P\bar{6}m2$ (187)	*a* = 3.32 Å<br>*b* = 3.32 Å<br>*c* = 20 Å	Mo 1*f* (0.33333, 0.66667, 0.5)<br>Se 2*h* (0.66667, 0.33333, 0.41656)

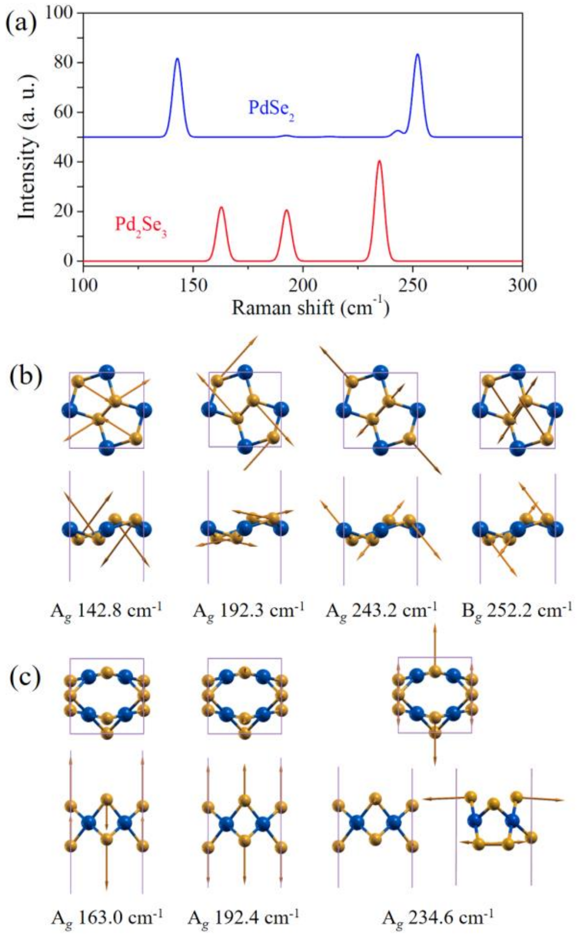

Figure S1. (**a**) Raman spectra of $PdSe_2$ and Pd_2Se_3 monolayers. (**b**) and (**c**) are the corresponding Raman-active vibrational modes of the two structures.

Raman spectroscopy as one of efficient characterization techniques has been widely used to distinguish newly synthesized materials. Here, using the density functional perturbation theory as implemented in the Quantum ESPRESSO package [1], we calculated the Raman spectra of Pd_2Se_3 and $PdSe_2$ for future experimental characterization. The calculated results are presented in Figure S1a, which shows that there are four peaks in the Raman spectra of $PdSe_2$, including two strong peaks at 142.8 (A_g) and 252.2 (B_g) cm^{-1} and two weak peaks at 192.3 (A_g) and 243.2 (A_g) cm^{-1}. Further analyzing the atomic vibrations, we note that the four Raman-active modes are mainly for the movements of Se-Se dumbbells, as indicated in Figure S1b. In contrast, the Raman spectra of Pd_2Se_3 displays three distinct peaks, with Raman shift centered at 163, 192.4, and 234.6 cm^{-1} respectively. These three Raman-active modes all belong to A_g symmetry. However, their atomic vibrations in Figure S1c show that the first two modes involve the movements of both the Se–Se dumbbells and the unpaired Se atoms, while the highest mode solely results from the vibrations of Se–Se dumbbells.

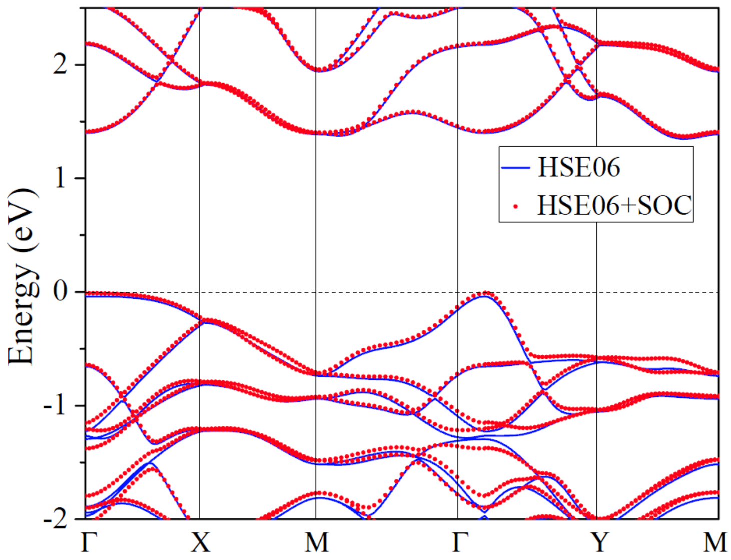

Figure S2. Electronic band structure of the Pd_2Se_3 monolayer calculated at the HSE06 level with and without considering the SOC interaction.

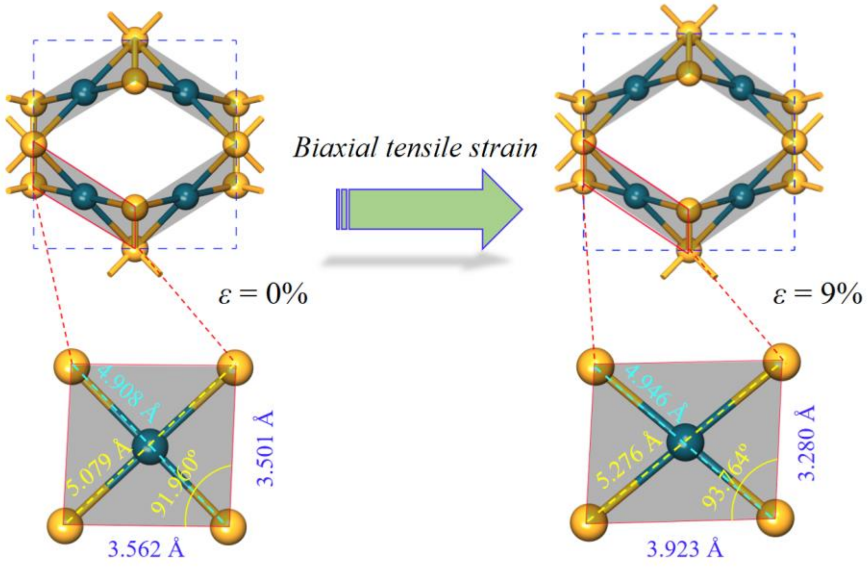

Figure S3. Geometric structure of the Pd_2Se_3 monolayer under 0% and 9% biaxial tensile strain.

In the Pd_2Se_3 monolayer, the Pd atom is coordinated by four Se atoms, forming the ($PdSe_4$) units of the structure. For the unstrained structure in the left panel of Figure S3, the ($PdSe_4$) unit has a nearly perfect square-planar geometry, with small distortions caused by the different chemical environments of Se atoms. Nevertheless, since the deviation from the perfect geometry is rather small, the splitting of Pd 4*d* orbitals can be treated as in a standard square-planar crystal filed. However, once the biaxial tensile strain is applied to the structure, the Pd-Se bond lengthens and more obvious distortions are observed as shown in the right panel of Figure S3. These structural changes are responsible for the reduced energies of the valence and conduction bands (antibonding states) and the increased bandgaps of Pd_2Se_3 under biaxial tensile strains.

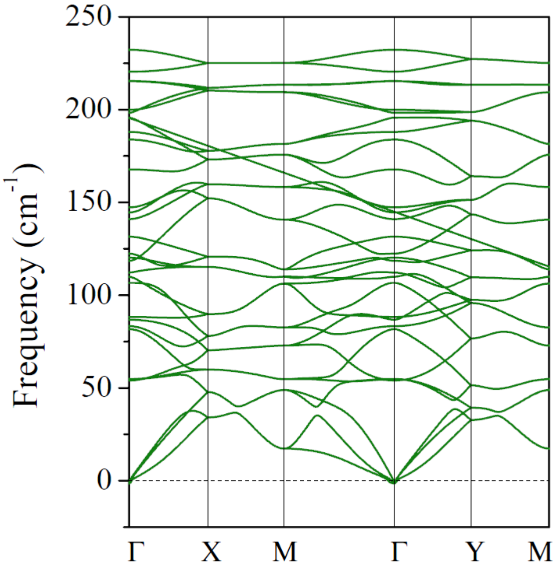

Figure S4. Phonon dispersion of the Pd_2Se_3 monolayer under 9% biaxial tensile strain.

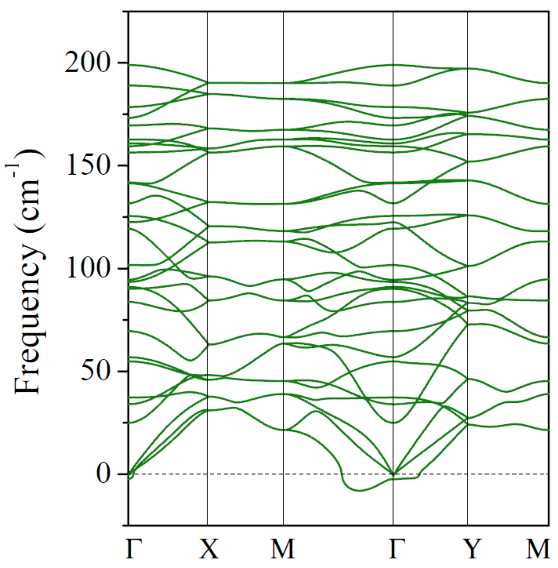

Figure S5. Phonon dispersion of the Pd_2Te_3 monolayer.

References

1. Paolo, G.; Stefano, B.; Nicola, B.; Matteo, C.; Roberto, C.; Carlo, C.; Davide, C.; Guido, L. C.; Matteo, C.; Ismaila, D.; Andrea Dal, C.; Stefano de, G.; Stefano, F.; Guido, F.; Ralph, G.; Uwe, G.; Christos, G.; Anton, K.; Michele, L.; Layla, M.-S.; Nicola, M.; Francesco, M.; Riccardo, M.; Stefano, P.; Alfredo, P.; Lorenzo, P.; Carlo, S.; Sandro, S.; Gabriele, S.; Ari, P. S.; Alexander, S.; Paolo, U.; Renata, M. W. Quantum Espresso: A Modular and Open-Source Software Project for Quantum Simulations of Materials. *J. Phys. Condens. Matter* **2009**, *21*, 395502.
